# Labor-Saving

**Published:** 1881-01

**Authors:** Thos. K. Beecher


### LABOR-SAVING.

THOS. K. BEECHER.

To get on and get up so as to be beyond the necessity of work, is the aim of nearly every man I meet. And since he who has money in hand can buy the services of man and beast, we are pushing for money, and dreaming or longing every day for that good time, when we shall not have to work. Mechanics, farmers and laboring men generally eye the houses, and horses, and coaches and clothes of the well-to-do, and *wish*—if they do not envy. And when success has been earned by industry, men in all ages turn away from labor—physical labor, and shout with gladness at the advent of any labor-saving device. Thus, I may say in a general way, that men are more nearly unanimous in keeping away from work and wishing to be rid of it forever than they are to escape from vice or wickedness.

It goes without saying—everybody knows—that there is a severity of toil that stunts the growth, shadows the cheer, stiffens the joints, enfeebles the intellect, benumbs the affections, and shortens the lives, of men. But all men have not noted that at the opposite extreme, when men go free from compulsory toil they suffer perhaps a worse disaster. Labor-saving, whether it comes to us by the generosity of nature or by the achievements of human ingenuity and co-operation, is not necessarily a blessing to the receiver.

There be sterile northern latitudes where human nature is dwarfed down to the stature and stupidity of the Lapps and Esquimaux. Equally there be genial skies and food bearing soils which gratify men with unearned gifts, until they cease to be men in any proper sense. They become sleek but not sinewy, passionate but not not intelligent, superstitious, lazy, cruel. The temperate, wheat-growing zone of the globe is the zone of manhood and its highest achievements.

But I note along this zone, that men of the present age, by their industry, ingenuity and cooperations are manufacturing artificially a labor-saving society. They triumph by harnessing the forces of nature to their productive machinery. *Steam* works, and men sit by to enjoy the produce. I note also, that as a general rule, this artificial plenty, resulting in a manumission from compulsory toil brings to pass, or is bringing to pass the same evils that on the scale of nature we detect and disapprove among the Lapps at one extreme, and the Africans and South-sea islanders at the other. The "lucky" and undriven become sleek but not sinewy; passionate but not intelligent; superstitious, lazy, cruel; while the prolific lower classes sink down—down toward an aborted, stupid, playless brutality. In short, our blessed temperate zone is sending a portion of its people toward the voluptuous tropics, another portion toward the chill and dark of the polar circles.

Give me neither poverty nor riches, is the prayer of wisdom. May I never, never see the day when I am excused from sweaty toil. Equally afar be the days when necessity so imprisons me that I come short of the play of every faculty that goes to make up that grand creature of God—A HEALTHY MAN!

Labor-saving devices! How plausibly they come in upon us to our utter ruin. The spring is a hundred paces from the settler's house. Ten times a day the mother's call is heard—"fetch me a pail of water!" and the boy or girl bare-footed brings it. By and by a pipe is laid. Those trips to the spring gave fresh air, sunshine, exercise, health. How convenient to have running water in the kitchen! The boy or girl can go to school now! Yes, but if the good that came by "fetching water" fail to be supplied in some other way, the gain of the brain is too costly, if it have enfeebled legs, arms, lungs and the elasticity of physical life.

Of course every dairyman and farmer will lay pipe and lighten woman's work at his earliest ability. There ought to be a labor-saving process *begun*. But the question is, how far to carry this process.

Let twenty prosperous years go by. The sons of this farmer sell out and realize cash. They drift city-ward with their capital. Taking brains into partnership they set up a prosperous bank and *earn* (so they call it,) ten per cent. every year. Now let us see these country boys enjoying labor-saving city devices. Let clothing and jewels pass by uncriticised and undeplored. WHAT CAN THESE BOYS DO?

Horses and coachmen excuse from walks. A janitor sweeps and warms the steam-heated office. A telegraph summons an errand boy; a telephone speaks all over the city and orders marketing and dinner. A street car shelters from showers and snow. A carrier brings the mail. A barber shaves and combs the hair. A porter blacks and warms the dainty shoes. A valet, or a wife or wife's maid lays out and airs the linen and brushes garments. Wine or hot whiskey and cigars keep up a semblance of thought Hired singers warble for him, paid professors perform. Artizans called by telephone keep the home in repair.

And this man—this MAN—a farmer's son fattens, softens, stupefies until he becomes a helpless blob of imbecility, able only to cry out at danger and offer to pay for a strong government to protect his property!

There has been too much labor saving—that's all. He was a working man. By labor-saving he has become the helpless, worthless button that glitters useless on the showy front of our civilization.

And yet our untaught or mistaught boys and girls are longing and thinking, and by help of our schools are trying to get up in society to where they'll not have to work for a living!

Three searching questions shall end this paper.

1. Do you, reader! see that labor is a blessing and not a curse?

2. Do you honor and admire contented, honest working people?

3. Are you yourself working and teaching your children "to work with their hands."

If not, why not?

